# Coarse Initial Orbit Determination for a Geostationary Satellite Using Single-Epoch GPS Measurements

**DOI:** 10.3390/s150407878

**Published:** 2015-04-01

**Authors:** Ghangho Kim, Chongwon Kim, Changdon Kee

**Affiliations:** School of Mechanical and Aerospace Engineering and SNU-IAMD Seoul National University, 1 Gwanak-ro Gwanak-gu, Seoul 151-744, Korea; E-Mails: chew79@snu.ac.kr (G.K.); nan772@snu.ac.kr (C.K.)

**Keywords:** GEO, GPS, initial state, orbit determination, EKF, space GPS receiver

## Abstract

A practical algorithm is proposed for determining the orbit of a geostationary orbit (GEO) satellite using single-epoch measurements from a Global Positioning System (GPS) receiver under the sparse visibility of the GPS satellites. The algorithm uses three components of a state vector to determine the satellite’s state, even when it is impossible to apply the classical single-point solutions (SPS). Through consideration of the characteristics of the GEO orbital elements and GPS measurements, the components of the state vector are reduced to three. However, the algorithm remains sufficiently accurate for a GEO satellite. The developed algorithm was tested on simulated measurements from two or three GPS satellites, and the calculated maximum position error was found to be less than approximately 40 km or even several kilometers within the geometric range, even when the classical SPS solution was unattainable. In addition, extended Kalman filter (EKF) tests of a GEO satellite with the estimated initial state were performed to validate the algorithm. In the EKF, a reliable dynamic model was adapted to reduce the probability of divergence that can be caused by large errors in the initial state.

## 1. Introduction

Many ground-tracking networks and facilities are required to track the position of geostationary orbit (GEO) satellites if a ground-tracking system is used [1,2]. The Global Positioning System (GPS) receiver can provide high-accuracy position data at a low cost; thus, it is reasonable to use GPS receivers in GEO satellites. However, position accuracy, which is calculated using a single-point solution (SPS) algorithm with snapshot measurements, is low compared with that of low Earth orbit (LEO) satellites or ground users; in certain cases, no result is produced. These disadvantages occur because the GPS signal power is lower than that of the LEO satellite, and the geometrical configuration of the GPS satellites relative to the GEO satellites is unfavorable. Therefore, an orbit determination (OD) filter is needed to overcome these unfavorable circumstances [3].

There are two main types of orbit determination (OD) filters: post-processing and real-time processing techniques. A real-time OD filter is needed to operate a geostationary orbit (GEO) satellite instantly and properly; one such filter is the extended Kalman filter (EKF), which is well known for its accuracy and efficiency. The EKF convergence time is determined by the initial state and conditions [4]. However, most studies do not consider how to determine the initial state and its conditions [5]. More than four GPS satellites are observable in low Earth orbit (LEO); thus, single-point solutions (SPS) can be applied at any time and the result can be used as the initial states of the EKF with a high level of accuracy. Typically, fewer than four GPS satellites are observable at the GEO; therefore, SPS are not always applicable. In these situations, alternative solutions must be employed, such as the short arc batch technique, which is a post-processing technique that requires measurements over a long period of time; thus, this technique does not provide navigation data instantly [6,7].

**Figure 1:**
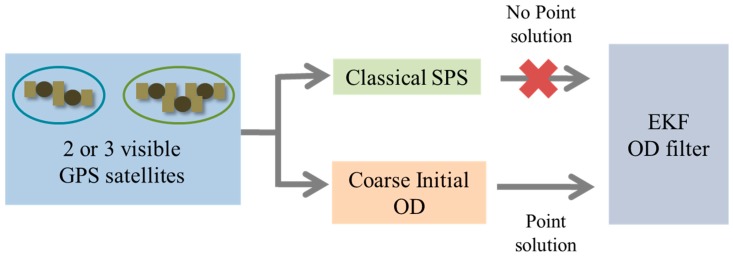
Application of the coarse initial orbit determination algorithm.

In this paper, we developed a coarse initial orbit determination algorithm to improve the accuracy of the initial EKF states under the sparse visibility of GPS satellites. The application of the proposed algorithm is illustrated in Figure 1. We used the characteristics of GEOs to develop our algorithm: GEO is an almost circular orbit, and its inclination angle is nearly zero. We set the minimum number of state components to calculate the state of the satellite using snapshot measurements under the sparse visibility of GPS satellites. This algorithm can determine the GEO satellite’s state vector even when fewer than four GPS satellites are visible, and it is very practical because it does not require long-term measurements.

The remainder of this paper is organized as follows. In Section 2, the details of the coarse initial orbit determination algorithm are explained. In Section 3, the general EKF-based orbit determination scheme is discussed. In Section 4, simulations of the proposed algorithm and EKF are performed to test the accuracy and the availability of the proposed algorithm. Finally, the results of the developed algorithm and EKF are discussed.

## 2. Coarse Initial Orbit Determination Algorithm

Four unknown variables appear in the classical SPS algorithm using the GPS signal: position components (x,y,z) and receiver clock bias error (${\text{δ}}_{r}$). Thus, measurements from at least four GPS satellites are required. The user position can typically be calculated using the GPS at a LEO satellite at any time because more than four GPS satellites are observed at the LEO satellite’s altitude. The error of the calculated single point position of a LEO is less than several dozens of meters and can be used as the initial state of the EKF. However, it is impossible to calculate the position using the classical SPS algorithm when fewer than four GPS satellites are visible at the GEO. More than four GPS satellites are infrequently visible at the GEO satellite; thus, the point position is not always determined. Thus, we must wait until more than four GPS satellites are visible to obtain navigation data.

**Figure 2:**
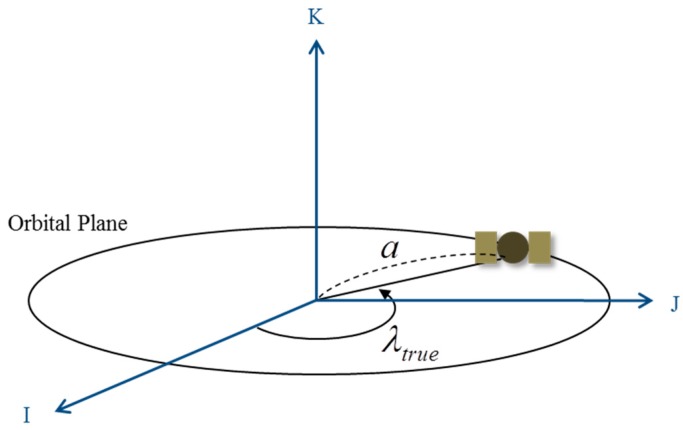
Simplified geostationary orbit (GEO) model. a = 42, 164 km and *e* = *i* = 0, *e* is the eccentricity and *i* is the inclination.

We developed a coarse initial orbit determination algorithm to calculate a point solution using measurements obtained from the receiver with two or three GPS satellites. The algorithm uses a minimum number of state variables, which were selected by considering the characteristics of the GEO. The classical orbital elements of the ideal GEO are shown in Figure 2.

The right ascension of the ascending node (RAAN) of the ideal GEO cannot be defined because the inclination angle is zero. The eccentricity is also zero, and thus, the argument of perigee cannot be defined either. Thus, we can define the state of the GEO satellite using only its true longitude value. The true longitude is a useful term when defining circular and equatorial orbits, and its equation is given by

(1) $$\cos\left({\text{λ}}_{true}\right)=\frac{\hat{I}\cdot \vec{r}}{\left|\hat{I}\right|\left|\vec{r}\right|}$$

If we assume that the satellite rotates in the ideal GEO, the states of a satellite can be approximately expressed by one element: the true longitude. We included two additional variables (the clock bias and clock bias rate of the receiver) in the state vector of the GEO satellite because these error components are included in the C/A code pseudorange and pseudorange rate. Thus, the state vector of the GEO satellite is defined as

(2) $$X_{Kep}={\left[\begin{array}{ccc} {\text{λ}}_{true} & c{\text{δ}}_{r} & c{\dot{\text{δ}}}_{r} \end{array}\right]}^{T}$$

where c is the speed of light, ${\text{δ}}_{r}$ is the receiver’s clock bias, and ${\dot{\text{δ}}}_{r}$ is the receiver’s clock bias rate.

The state vector in Equation (2) can be converted into Cartesian coordinates and given by

(3) $$X_{cart}={\left[\begin{array}{cccccccc} a\cos({\text{λ}}_{true}) & a\sin({\text{λ}}_{true}) & 0 & -a\sin({\text{λ}}_{true}){\dot{\text{λ}}}_{true} & a\cos({\text{λ}}_{true}){\dot{\text{λ}}}_{true} & 0 & c{\text{δ}}_{r} & c{\dot{\text{δ}}}_{r} \end{array}\right]}^{T}$$

(4) $${\dot{\text{λ}}}_{true}=\dot{M}=n=\sqrt{\frac{GM_{⊕}}{a^{3}}}$$

where G is the gravitational constant and $M_{⊕}$ is the mass of the Earth. The value of a was set to a constant, and thus, the differential of a is zero.

Then, the measurement vector can be defined as

(5) $$Z={\left[\begin{array}{ccccc} {\text{ρ}}_{1} & {\dot{\text{ρ}}}_{1} & \cdots & {\text{ρ}}_{M} & {\dot{\text{ρ}}}_{M} \end{array}\right]}^{T}$$

(6) $$\text{ρ}=\left|{\vec{r}}_{t}-\vec{r}\right|-c{\text{δ}}_{t}+c{\text{δ}}_{r}+n$$

(7) $$\dot{\text{ρ}}=\hat{e}\cdot \left({\vec{v}}_{t}-\vec{v}\right)-c{\dot{\text{δ}}}_{t}+c{\dot{\text{δ}}}_{r}+n$$

(8) $$\hat{e}=\frac{{\vec{r}}_{t}-\vec{r}}{\left|{\vec{r}}_{t}-\vec{r}\right|}$$

where M is the number of visible GPS satellites (two or three in this paper); ${\vec{r}}_{t}$ and ${\vec{v}}_{t}$ are the position and velocity vectors, respectively, of the GPS satellite; $\vec{r}$ and $\vec{v}$ are the position and velocity vectors, respectively, of the GEO satellite; ${\text{δ}}_{t}$ and ${\dot{\text{δ}}}_{t}$ are the GPS satellite clock bias and clock bias rate, respectively; ${\text{δ}}_{r}$ and ${\dot{\text{δ}}}_{r}$ are the receiver clock bias and clock bias rate, respectively; $\hat{e}$ is the unit direction vector; ρ and $\dot{\text{ρ}}$ are the pseudorange and pseudorange rate, respectively; and n is the measurement noise from the assumed Gaussian distribution. The ionospheric error is not included in Equations (6) and (7) because we assume that the ionospheric error can be removed by the dual-frequency GPS receiver.

We use a least-squares technique to calculate $X_{Kep}$; this technique minimizes the square sum of the difference between Z and $Z_{ref}$ Z is the measurement vector obtained from the GPS receiver at the true point $X_{Kep}$, and $Z_{ref}$ is the calculated measurement vector at the reference point $X_{ref}$. We assume that the GEO satellite’s position is approximately known when the GPS receiver starts its own signal processing; thus, $X_{ref}$ is chosen with an error of 1000 km in the first calculation and is updated iteratively until convergence is reached. The function of $Z_{ref}$ is nonlinear, and thus, $Z_{ref}$ must by linearizing at the point $X_{ref}$. The least-squares technique to determine is given by

(9) $$\text{Δ}X_{Kep}=X_{Kep}-X_{ref}$$

(10) $$\text{Δ}Z=Z-Z_{ref}$$

(11) $$Z_{ref}={\left[\begin{array}{ccccc} {\text{ρ}}_{ref1} & {\dot{\text{ρ}}}_{ref1} & \cdots & {\text{ρ}}_{refM} & {\dot{\text{ρ}}}_{refM} \end{array}\right]}^{T}$$

(12) $${\text{ρ}}_{ref}=\left|{\vec{r}}_{t}-{\vec{r}}_{ref}\right|-c{\text{δ}}_{t}+c{\text{δ}}_{r}$$

(13) $${\dot{\text{ρ}}}_{ref}=\hat{e}\cdot \left({\vec{v}}_{t}-{\vec{v}}_{ref}\right)-c{\dot{\text{δ}}}_{t}+c{\dot{\text{δ}}}_{r}$$

(14) $$\text{Δ}Z=H\text{Δ}X_{ref}$$

(15) $$\begin{array}{c} \text{Δ}X_{ref}={\left(H^{T}H\right)}^{-1}H^{T}\text{Δ}Z\\ H=H_{cart}H_{Kep} \end{array}$$

(16) $$X_{Kep}=X_{ref}+\text{Δ}X_{ref}$$

The equations of the measurement matrix $H_{Kep}$ and $H_{cart}$ are given by

(17) $$H_{Kep}=\frac{\partial X_{cart}}{\partial X_{Kep}}$$

(18) $$H_{cart}=\frac{\partial Z}{\partial X_{cart}}$$

If we define the state vector as Equation (2), we can calculate the position of the GEO satellite using single-epoch measurements of two or three GPS satellites. In the definition of the state vector in Equation (2), the inclination angle and eccentricity are not included in the variables; however, these variables exist in the real GEO orbit and could increase the error of the state vector calculated iteratively. The geometric state error estimated by the proposed algorithm can be defined as

(19) $$\left[\begin{array}{c} x\\ y\\ z \end{array}\right]=R_{3}(-\text{Ω})R_{3}(-i)R_{3}(-\text{ω})\left[\begin{array}{c} r\cos(\text{ν})\\ r\sin(\text{ν})\\ 0 \end{array}\right]$$

(20) $${\text{ε}}^{2}={\left(x-a\cos({\text{λ}}_{est})\right)}^{2}+{\left(y-a\sin({\text{λ}}_{est})\right)}^{2}+{\left(z\right)}^{2}+{\left(c{\text{δ}}_{r}-c{\text{δ}}_{est}\right)}^{2}$$

where Ω is the ascending node, i is the inclination, ω is the argument of perigee, ν is the true anomaly, r is the radius, ${\text{λ}}_{est}$ is the estimated true longitude, and $c{\text{δ}}_{est}$ is the estimated receiver clock bias.

In Equation (20), ${\left(z\right)}^{2}$ is a constant bias term that cannot be reduced or removed by the proposed algorithm; therefore, the error could be increased if the z-component has great value. However, we can still determine the state vector as accurately as possible given the sparse visibility of the GPS satellites. After calculating the state vector using Equations (9)–(18), the vector can be used as the initial state value for the EKF with a practical level of accuracy.

## 3. EKF Scheme

The EKF is well known for its accuracy and speed; thus, it is used in non-linear system applications, such as real-time OD. We will not explain the entire algorithm in detail, as it is not the focus of this paper. Thus, the equations of the EKF algorithm scheme are summarized in Table 1 [5,8].

**Table 1:** Extended Kalman filter (EKF) processing scheme using GPS measurements.

**Nonlinear Dynamics Model** $X_{k}={\text{ϕ}}_{k-1}(X_{k-1})+w_{k-1},\;w_{k}∼N(0,Q)$ **Nonlinear Measurement Model** $Z_{k}=h_{k}(X_{k})+v_{k},\;v_{k}∼N(0,R)$ **State and Measurement** $X={\left[\begin{array}{cccccccc} x & y & z & \dot{x} & \dot{y} & \dot{z} & c{\text{δ}}_{r} & c{\dot{\text{δ}}}_{r} \end{array}\right]}^{T}$ $Z={\left[\begin{array}{ccccc} {\text{ρ}}_{1} & {\dot{\text{ρ}}}_{1} & \cdots & {\text{ρ}}_{M} & {\dot{\text{ρ}}}_{M} \end{array}\right]}^{T}$ $\text{ρ}=\left|{\vec{r}}_{t}-\vec{r}\right|+c{\text{δ}}_{r}-c{\text{δ}}_{t}$ $\dot{\text{ρ}}=\hat{e}\cdot \left({\vec{v}}_{t}-\vec{v}\right)+c{\dot{\text{δ}}}_{r}-c{\dot{\text{δ}}}_{t}$
**Time update** Predicted state estimation: ${\bar{X}}_{k}=\phi_{k-1}({\hat{X}}_{k-1})$ Linear approximation: $\Phi_{k-1}\approx \frac{\partial \phi_{k-1}}{\partial X}{|}_{X={\bar{X}}_{k-1}}$ Predicted covariance matrix: ${\bar{P}}_{k}=\Phi_{k-1}{\hat{P}}_{k-1}{\Phi_{k-1}}^{T}+Q_{k-1}$
**Measurement update** Predicted Measurement: ${\bar{Z}}_{k}=h_{k}({\bar{X}}_{k})$ Measurement matrix linearization: $H_{K}\approx \frac{\partial h_{k}}{\partial X}{|}_{X={\bar{X}}_{k}}$ Kalman gain computation: $K_{k}={\bar{P}}_{k}H_{k}^{T}{\left[H_{k}{\bar{P}}_{k}H_{k}^{T}+R_{k}\right]}^{-1}$ State estimation: ${\hat{X}}_{k}={\bar{X}}_{k}+K_{k}(Z_{k}-{\bar{Z}}_{k})$ Updating posteriori covariance matrix: ${\hat{P}}_{k}=\left[I-K_{k}H_{k}\right]{\bar{P}}_{k}$

We used two-body gravity and other perturbations to predict the state of the satellite. The equation of the general accelerations acting on a satellite is [5]:

(21) $$\ddot{\vec{r}}=-\frac{\text{μ}}{r^{3}}\vec{r}+{\vec{a}}_{perturbed}$$

where $\vec{r}$ is the position vector of the satellite in the Earth-centered inertial (ECI) coordinate system, μ is the gravitational constant of the Earth and ${\vec{a}}_{perturbed}$ is the other perturbed acceleration.

We included gravitational attraction by a nonspherical central body, third-body effects (Sun and Moon) and solar-radiation pressure in the perturbed accelerations [5,9]. The gravitational attraction by the nonspherical central body is expressed as:

(22) $$U=\frac{\text{μ}}{r}\left[1+\sum_{n=2}^{\infty }\sum_{m=0}^{n}{\left(\frac{R_{⊕}}{r}\right)}^{n}p_{n,m}\left[\sin({\text{ϕ}}_{sat})\right]\left\{C_{n,m}\cos(m{\text{λ}}_{sat})+S_{n,m}\sin(m{\text{λ}}_{sat})\right\}\right]$$

where $R_{⊕}$ is the radius of the Earth, p is the associated Legendre polynomials, ${\text{ϕ}}_{sat}$ is the latitude of the satellite, ${\text{λ}}_{sat}$ is the longitude of the satellite, and C and S are the gravitational coefficients.

The equation of the third-body effect is expressed as:

(23) $${\ddot{\vec{r}}}_{⊕sat}=-\frac{{\text{μ}}_{⊕}{\vec{r}}_{⊕sat}}{r_{⊕sat}}+{\text{μ}}_{3}\left(\frac{{\vec{r}}_{sat3}}{{r^{3}}_{sat3}}-\frac{{\vec{r}}_{⊕3}}{{r^{3}}_{⊕3}}\right)$$

where ${\text{μ}}_{⊕}$ is the gravitational constant of the Earth, ${\text{μ}}_{3}$ is the gravitational constant of the third body, ${\vec{r}}_{⊕sat}$ is the position vector pointing from the Earth to the third body, ${\vec{r}}_{sat3}$ is the position vector pointing from the satellite to the third body and ${\vec{r}}_{⊕3}$ is the position vector pointing from the Earth to the third body.

The acceleration by solar radiation pressure is included and is one of the significant accelerations acting on GEO satellites. The solar radiation pressure is expressed as:

(24) $${\vec{a}}_{SR}=-\frac{P_{SR}C_{R}A_{⊙}}{m}\frac{{\vec{r}}_{sat⊙}}{\left|{\vec{r}}_{sat⊙}\right|}$$

where $P_{SR}$ is the solar pressure, $C_{R}$ is the reflectivity, $A_{⊙}$ is the effective area of the satellite and m is the mass of the satellite.

We introduced some errors into the dynamics model of the EKF filter compared with the measurement dynamics model to better approximate a real situation, and the errors are listed in Table 2. The degree and order of the geopotential were lowered from 20 to 10, and the satellite area for the solar pressure model was adjusted to create a 10% error [2].

The errors of the initial state were assumed to be approximately 10 km and 0.01 km in position and velocity, respectively. With these values, we set the initial covariance as follows:

(25) $$P=diag\left[\begin{array}{cccccccc} {{\text{σ}}_{P1}}^{2} & {{\text{σ}}_{P1}}^{2} & {{\text{σ}}_{P1}}^{2} & {{\text{σ}}_{P2}}^{2} & {{\text{σ}}_{P2}}^{2} & {{\text{σ}}_{P2}}^{2} & {{\text{σ}}_{P1}}^{2} & {{\text{σ}}_{P2}}^{2} \end{array}\right]$$

where ${{\text{σ}}^{2}}_{P1}=10^{2}{\left(\text{km}\right)}^{2}$ and ${{\text{σ}}^{2}}_{P2}=10^{-4}{\left(\text{km}/\text{s}\right)}^{2}$.

The noise of the range and that of the range rate are Gaussian distributions with standard deviations of 0.01 km and 0.0001 km/s, respectively, in the simulation. We set R as follows for the case of two or three GPS satellites, respectively:

(26) $$R=diag\left[\begin{array}{cccc} {{\text{σ}}_{R1}}^{2} & {{\text{σ}}_{R2}}^{2} & {{\text{σ}}_{R1}}^{2} & {{\text{σ}}_{R2}}^{2} \end{array}\right]\;or\;diag\left[\begin{array}{cccccc} {{\text{σ}}_{R1}}^{2} & {{\text{σ}}_{R2}}^{2} & {{\text{σ}}_{R1}}^{2} & {{\text{σ}}_{R2}}^{2} & {{\text{σ}}_{R1}}^{2} & {{\text{σ}}_{R2}}^{2} \end{array}\right]$$

where ${{\text{σ}}^{2}}_{R1}=10^{-4}{\left(\text{km}\right)}^{2}$ and ${{\text{σ}}^{2}}_{R2}=10^{-8}{\left(\text{km}/\text{s}\right)}^{2}$.

**Table 2:** COMS orbital elements.

	Measurement Dynamics	EKF Dynamics Error
Initial epoch time	UTC 00:00:00 1 January 2006	-
Simulation time	24 h	-
Geopotential model	EGM-96 (Degree: 20, Order: 20)	(Degree: 10, Order: 10)
Third-body gravity	Sun, Moon (DE405)	-
Solar pressure	4.57 × 10^−6^ N/m^2^	-
Cross-sectional area	18.941 m^2^	17.046 m^2^ (10% error)
Satellite mass	1547 kg	-
Numerical integration algorithm	Runge-Kutta 68	-
X	−27,828.9136 (km)	-
Y	−31,685.02205 (km)	-
Z	3.51107 (km)	-
V_x_	2.30981 (km/s)	-
V_y_	−2.02866 (km/s)	-
V_z_	−0.00192 (km/s)	-

The elements of the process noise covariance must be approximately 10^−16^, with the same level of error as the dynamics model of the filter; however, we tuned these values with consideration for the initial state error and convergence time [10,11]. We determined Q as follows:

(27) $$Q=diag\left[\begin{array}{cccccccc} {{\text{σ}}_{Q1}}^{2} & {{\text{σ}}_{Q1}}^{2} & {{\text{σ}}_{Q1}}^{2} & {{\text{σ}}_{Q2}}^{2} & {{\text{σ}}_{Q2}}^{2} & {{\text{σ}}_{Q2}}^{2} & {{\text{σ}}_{Q1}}^{2} & {{\text{σ}}_{Q2}}^{2} \end{array}\right]$$

where ${{\text{σ}}^{2}}_{Q1}=10^{-12}{\left(\text{km}\right)}^{2}$ and ${{\text{σ}}^{2}}_{Q2}=10^{-16}{\left(\text{km}/\text{s}\right)}^{2}$.

## 4. Simulation and Results

### 4.1. Simulation Procedure

**Figure 3:**
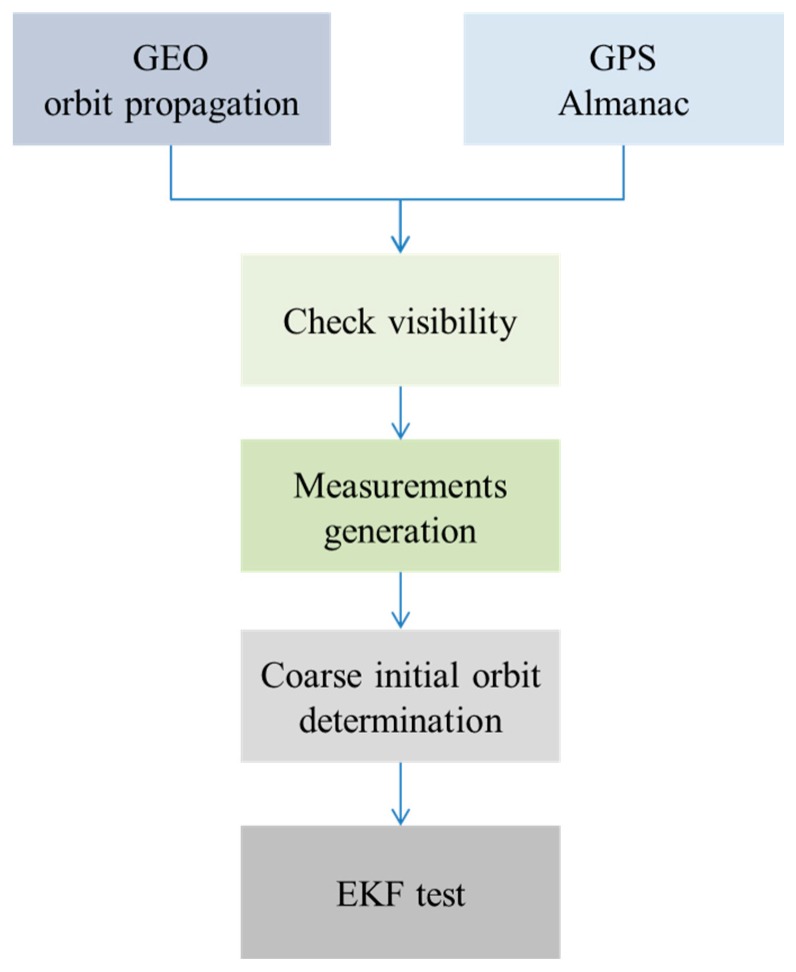
Diagram of the simulation procedure.

We chose the Communication, Ocean and Meteorological Satellite (COMS) launched by the Korea Aerospace Research Institute (KARI) as our GEO satellite for simulation. The COMS is located at 128.2° east, and COMS missions are related to Ka-band communication services, meteorological monitoring, and ocean observation [2]. We simulated the satellite’s orbit to validate and test the developed algorithm. First, we generated the position and velocity data of COMS for 24 h using a numerical orbit propagation algorithm. Then, the pseudorange and pseudorange rate, which were obtained from the receiver in the GEO satellite, were generated with simulated GPS signals. The developed algorithm was tested using the generated measurements under the condition that two or three GPS satellites were observable. Then, the calculated state vector was set to the initial state of the EKF, and the EKF was processed. The overall simulation procedure is summarized in Figure 3.

**Figure 4:**
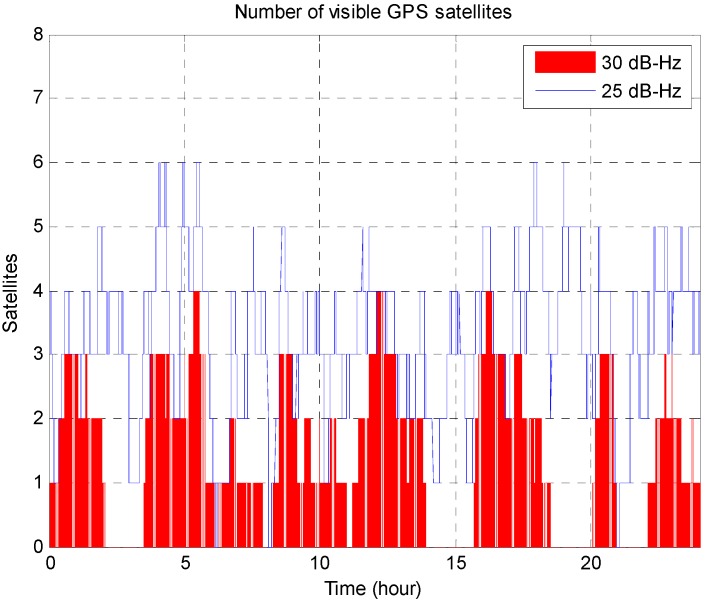
Number of visible GPS satellites over 24 h with 25 dB-Hz and 30 dB-Hz thresholds for signal acquisition and tracking; 30 dB-Hz is the minimum value that conventional receivers can track.

### 4.2. Generation of Measurements

The first simulation step was to propagate the GEO satellite’s orbit. We propagated the GEO using Cowell’s method, which propagates the position and velocity of the satellite by integrating the accelerations caused by perturbations at each time step [5]. We included the geopotential, solar pressure, and third-body gravity (the Sun and Moon). We chose the EGM-96 model as the geopotential, and the degree and order were both set to 20. The Runge-Kutta 68 algorithm was chosen as the numerical integrator, and the integral step was set to 10 s. The initial orbital elements for propagation are listed in Table 2.

The GPS satellites’ position and velocity were generated every 10 s for 24 h using the Almanac data. After generating the positions and velocities of the GEO and GPS satellites, we checked whether the GPS satellites were observable at each epoch. A GPS satellite was only visible when it was not blocked by the Earth and its signal power was sufficiently strong to be processed by the GPS receiver.

After determining the visibility of the GPS satellites, the C/A code pseudorange and pseudorange rate were calculated. The C/A code pseudorange is given by [12]

(28) $$\begin{array}{c} \text{ρ}=\left|{\vec{r}}_{t}(TX)-\vec{r}(RX)\right|+c{\text{σ}}_{r}(RX)-c{\text{σ}}_{t}(TX)+n\\ =\left|{\vec{r}}_{t}(RX-\text{τ})-\vec{r}(RX)\right|+c{\text{σ}}_{r}(RX)-c{\text{σ}}_{t}(RX-\text{τ})+n \end{array}$$

(29) $$\begin{array}{c} {\text{σ}}_{r}=b_{0}+b_{1}\left(t-t_{0}\right)\\ {\text{σ}}_{t}=a_{0}+a_{1}\left(t-t_{0}\right) \end{array}$$

where ρ is the C/A code pseudorange in L1, RX is the signal reception time, TX is the signal emission time, ${\text{σ}}_{r}$ is the receiver clock bias, ${\text{σ}}_{t}$ is the GPS satellite clock bias, n is noise, τ is the time delay, $t_{0}$ is the reference time, $a_{0}$ and $a_{1}$ are the polynomial coefficients of the GPS satellite clock bias, and $b_{0}$ and $b_{1}$ are the polynomial coefficients of the receiver clock bias.

The geometric distance from the GPS satellite to the receiver was calculated using ${\vec{r}}_{t}(TX)$ and $\vec{r}(RX)$; we did not use $\vec{r}(TX)$ [13]. The GPS signal travels through space at the speed of light, and thus, the receiver does not instantly receive the signal emitted from the GPS satellite. Therefore, the signal reception time is later than the signal emission time. The equation of the elapsed time from the emission to reception is given by

(30) $$\begin{array}{c} \text{τ}=\frac{\left|{\vec{r}}_{t}(TX)-\vec{r}(RX)\right|}{c}\\ =\frac{\left|{\vec{r}}_{t}(RX-\text{τ})-\vec{r}(RX)\right|}{c} \end{array}$$

τ appears on both sides of Equation (30). Thus, an iteration technique was used to calculate the proper τ. First, ${\vec{r}}_{t}(RX)$ was used instead of ${\vec{r}}_{t}(RX-\text{τ})$ to calculate the temporary τ on the left side of the equation, Then, the temporary τ was used on the right side of Equation (30) to update the temporary τ, and the iterations continued until τ converged [13,14].

The equation for the pseudorange rate is similar to that for the pseudorange and is given by

(31) $$\dot{\text{ρ}}=\hat{e}\cdot \left[{\vec{v}}_{t}(TX)-\vec{v}(RX)\right]+c{\dot{\text{δ}}}_{r}(RX)-c{\dot{\text{δ}}}_{t}(TX)+n$$

where ${\dot{\text{δ}}}_{r}$ and ${\dot{\text{δ}}}_{t}$ are the clock bias rates of the receiver and GPS satellite, respectively. These variables are calculated from the derivatives of ${\text{σ}}_{r}$ and ${\text{σ}}_{t}$.

### 4.3. Simulation Results

The algorithm was tested using data from the four points (A, B, C and D) selected from the 24 h simulated GEO orbit. We selected four points at 6 h intervals to rigorously validate the algorithm. The simulation times of the selected points A, B, C and D were UTC 00:00:00, 06:00:00, 12:00:00 and 18:00:00, respectively, on 1 January 2006. The position and velocity vector of each point is given in Table 3.

**Table 3:** Position and velocity of the COMS at the four selected points.

Point	A	B	C	D
UTC time	00:00:00	06:00:00	12:00:00	18:00:00
x (km)	−27,828.9136	31,792.4080	27,551.1020	−32,042.5366
y (km)	−31,685.0220	−2769.1326	31,911.9004	27,494.4159
z (km)	3.5110	−27.4958	−5.3747	28.3683
v_x_ (km/s)	2.3098	2.0194	−2.3276	−1.9989
v_y_ (km/s)	−2.0286	2.3185	2.0093	−2.3364
v_z_ (km/s)	−0.0019	−0.0003	0.0019	0.0004
Semi-major axis (km)	42,165.029	42,166.897	42,165.112	42,167.097
Eccentricity	0.000140	0.000108	0.000142	0.000192
Inclination (deg)	0.0360	0.0378	0.0378	0.0393
Ascending node (deg)	56.3009	58.8100	60.3141	61.9844
Argument of perigee (deg)	348.0949	325.9801	12.1476	17.0192
True anomaly (deg)	172.4067	260.1279	348.8802	77.4768

The observable GPS satellites over 24 h are depicted in Figure 4; however, the simulations were performed under controlled conditions in which only two or three GPS satellites were visible. The initial error in the reference position was set to 1000 km at each of the four points, and the reference receiver clock bias was set to 100 km. The noise terms in Equations (28) and (31) were selected from Gaussian distributions with standard deviations of 0.01 km and 0.0001 km/s, respectively.

**Figure 5:**
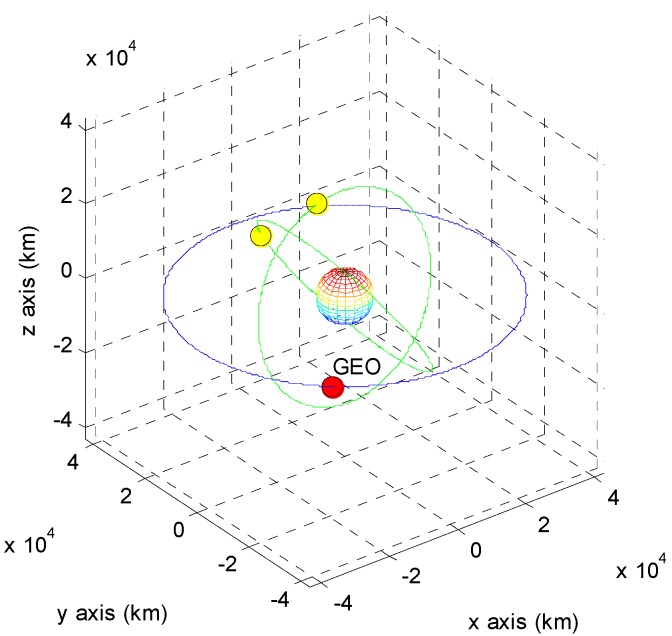
Point A with two visible GPS satellites.

**Figure 6:**
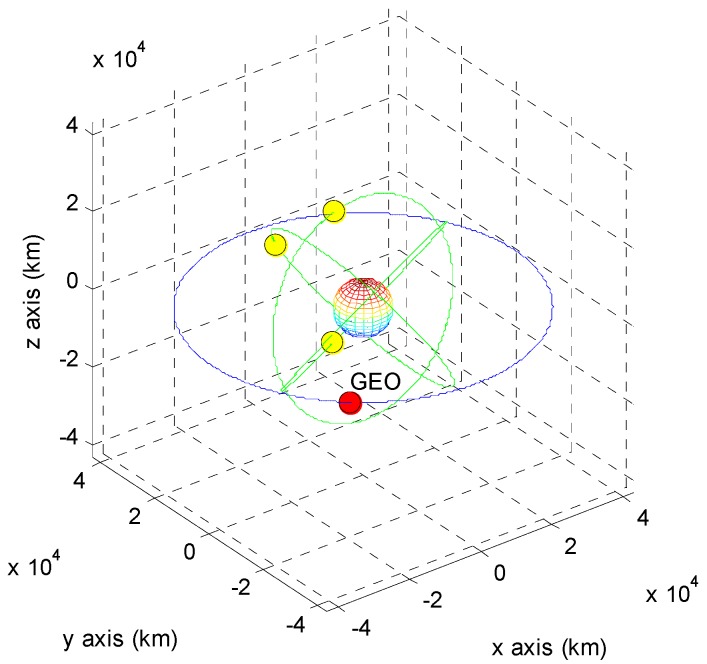
Point A with three visible GPS satellites.

**Figure 7:**
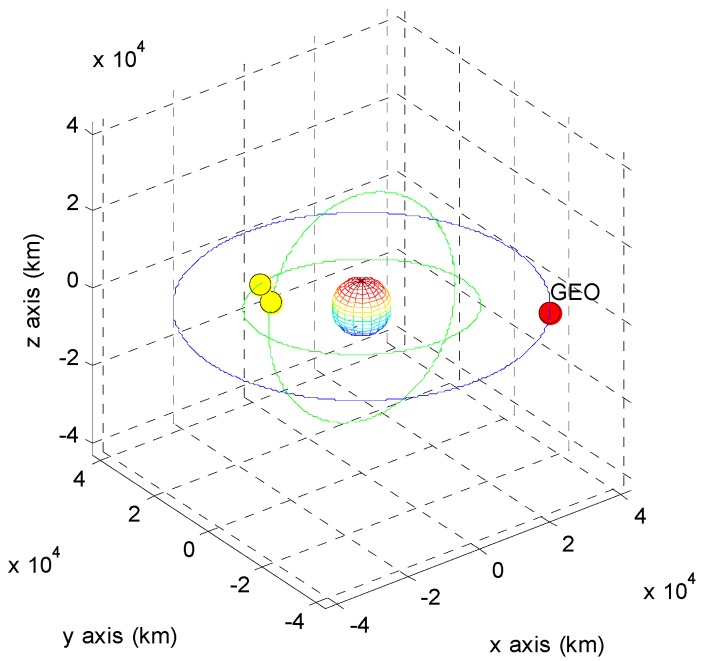
Point B with two visible GPS satellites.

**Figure 8:**
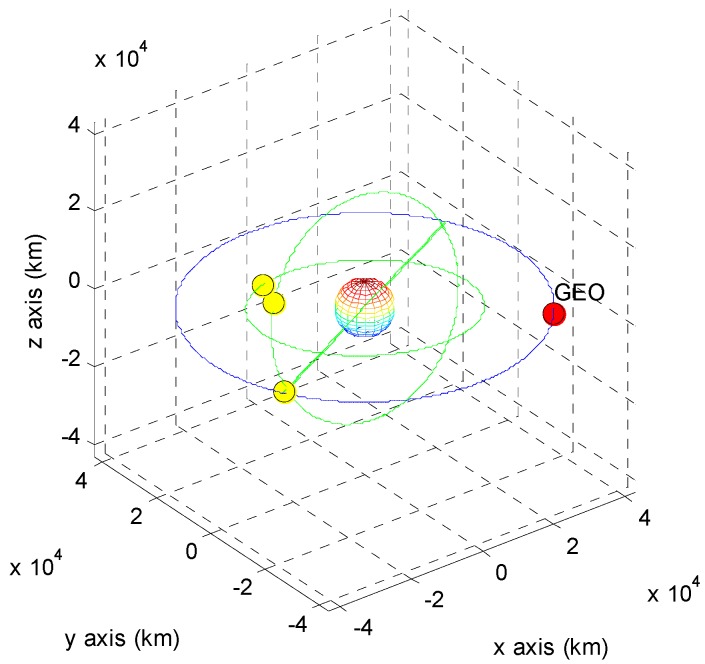
Point B with three visible GPS satellites.

**Figure 9:**
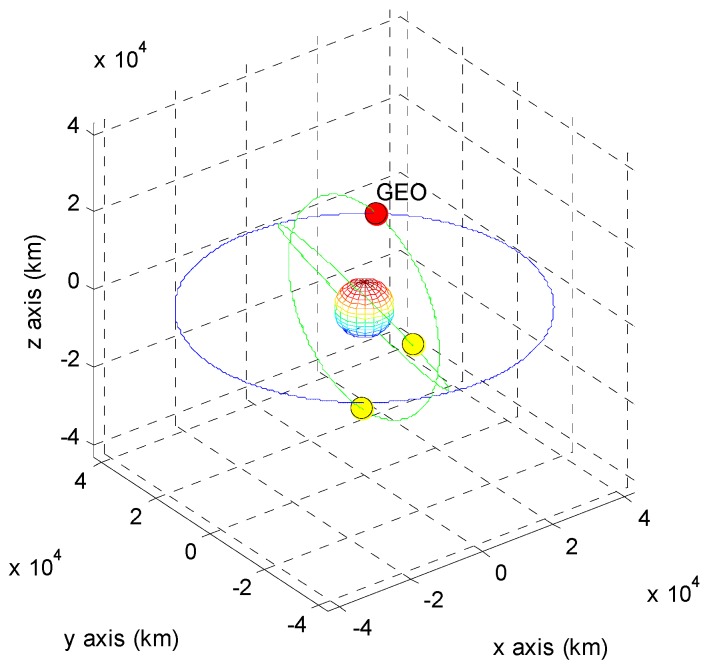
Point C with two visible GPS satellites.

**Figure 10:**
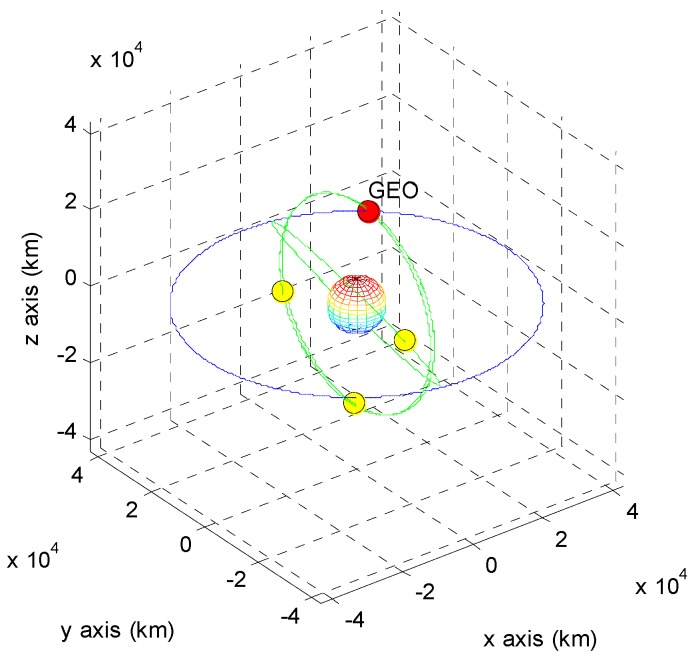
Point C with three visible GPS satellites.

**Figure 11:**
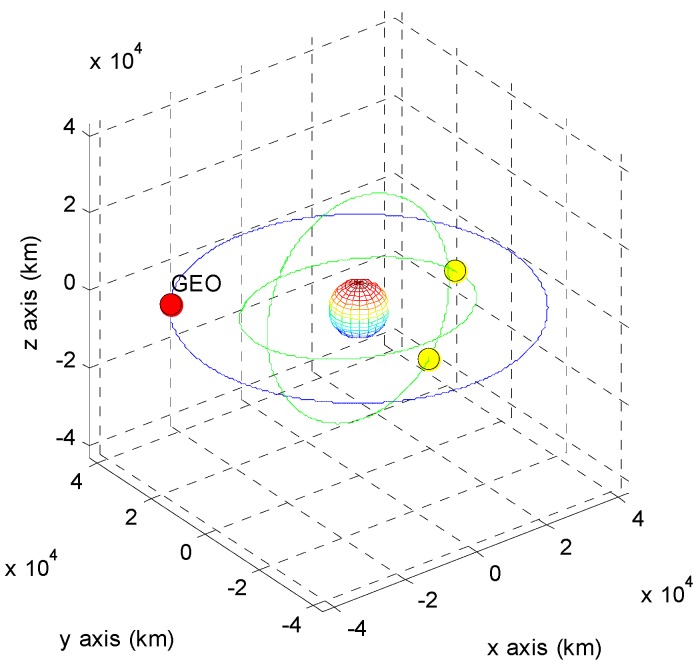
Point D with two visible GPS satellites.

**Figure 12:**
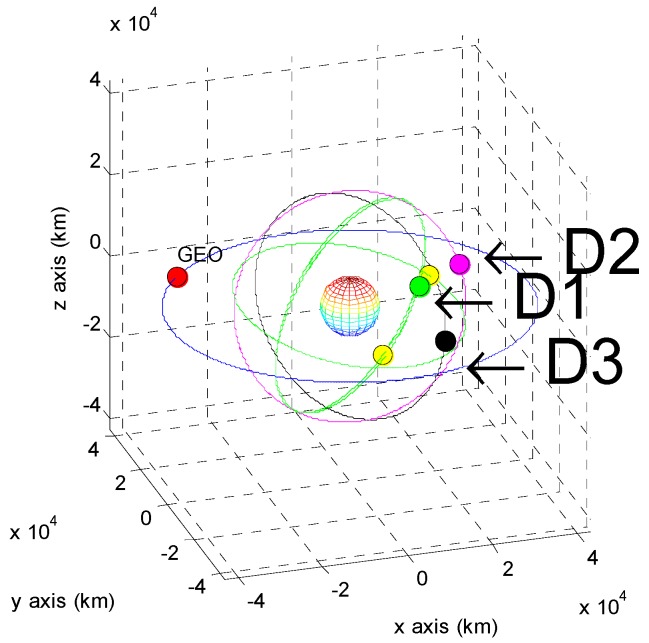
Point D with three visible GPS satellites.

The positions of the COMS and its visible GPS satellites at each point are shown in Figure 5, Figure 6, Figure 7, Figure 8, Figure 9, Figure 10, Figure 11 and Figure 12. The red spot represents the COMS, and the yellow spots represent GPS satellites. At each point, we produced scenarios such that two or three GPS satellites were visible at the COMS. Thus, we intentionally chose GPS satellites among those visible to control the number of visible satellites if more than three GPS satellites were visible. For consistency, we simply removed one satellite from the scenario where three GPS satellites were visible such that only two GPS satellites were visible. As shown in the figures, the visible GPS satellites are located behind the Earth and are located closely together.

We tested the developed algorithm using a single-epoch measurement at each point. The calculated state vectors of the COMS were compared to the true values, and the differences of each are summarized in Table 4 and Table 5. We also tested the influence of the position of the third GPS satellite at point D; more than three GPS satellites are observable at point D. We ran simulations with two fixed GPS satellites and a third satellite placed at several different positions. The results are summarized in Table 6. The residual refers to the difference between the calculated and true values. The results indicate that the residual of the calculated position is less than 40 km in range, and three of the four points have residuals of less than several kilometers in range when using three visible GPS satellites.

**Table 4:** Estimated error when using two visible GPS satellites.

Residuals	A	B	C	D
x (km)	4.453	13.415	−2.984	16.474
y (km)	5.113	16.693	8.600	20.910
z (km)	−3.511	27.495	5.374	−28.268
V_x_ (km/s)	0.000325	−0.000971	0.000001	−0.000963
V_y_ (km/s)	0.000331	0.000796	−0.000543	0.001067
V_z_ (km/s)	0.001920	0.000348	−0.001994	−0.000458
clock bias (km)	6.019	2.108	−5.550	−9.404
clock bias rate (km/s)	0.000001	−0.000519	−0.000276	0.000424

**Figure 13:**
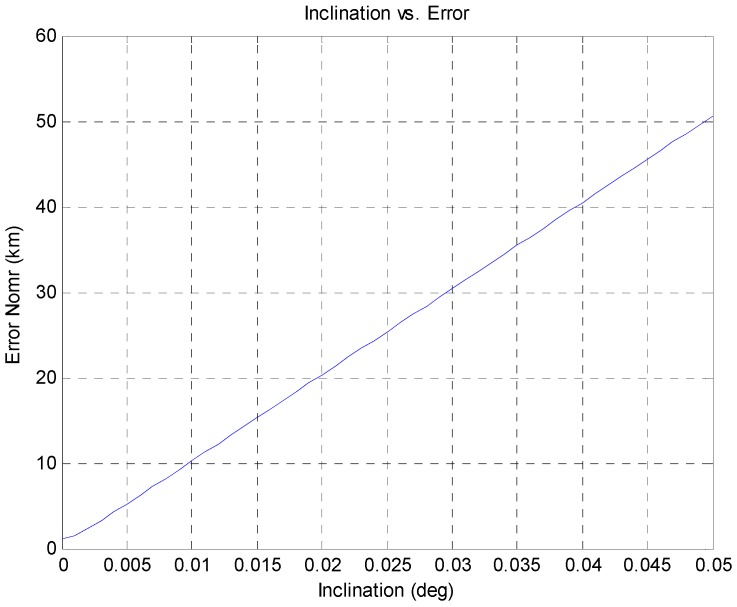
Relationship between inclination and the estimated error at point D.

The residuals increase as the z-component of orbital state increases. The maximum error occurs when the z-component of the orbital state is greatest, and the errors are small when the z-components of the orbital state are small. This relationship occurs because the developed algorithm does not include the z-component in the state vector, and thus, the z-component in the real orbit influences the x- and y-components in the state vector. The z-component value increases with the inclination and the relationship between the inclination and error at point D are presented in Figure 13. Based on Figure 13, we can conclude that the accuracy level of the proposed algorithm is high when the GEO satellite’s inclination is small.

**Table 5:** Estimated error when using three visible GPS satellites.

Residuals	A	B	C	D1
x (km)	1.908	4.642	4.951	17.425
y (km)	7.349	6.615	1.750	22.021
z (km)	−3.511	27.495	5.374	−28.268
V_x_ (km/s)	0.000162	−0.000236	0.000514	−0.001044
V_y_ (km/s)	−0.000517	0.000156	0.000003	0.001136
V_z_ (km/s)	0.001920	0.000348	−0.001994	−0.000458
clock bias (km)	7.156	−1.598	−4.319	−10.088
clock bias rate (km/s)	−0.000194	−0.000261	−0.000472	0.000194

**Table 6:** Estimated errors when using two fixed GPS satellites and a third satellite at various positions at point D.

Residuals	D1	D2	D3
x (km)	17.425	5.2519	−7.992
y (km)	22.021	7.799	−7.787
z (km)	−28.268	−28.268	−28.268
V_x_ (km/s)	−0.001044	−0.000007	0.001122
V_y_ (km/s)	0.001136	0.000249	−0.000716
V_z_ (km/s)	−0.000458	−0.000458	−0.000458
clock bias (km)	−10.088	−5.146	−0.468115
clock bias rate (km/s)	0.000194	−0.000050	−0.000165

No significant difference occurs when using measurements from two or three visible GPS satellites, except for point B, where the residual decreases when using the measurements from three GPS satellites. Furthermore, the geometric relationship among the GPS satellites and the GEO also affected the accuracy of the algorithm, as demonstrated by the simulation results for point D.

**Figure 14:**
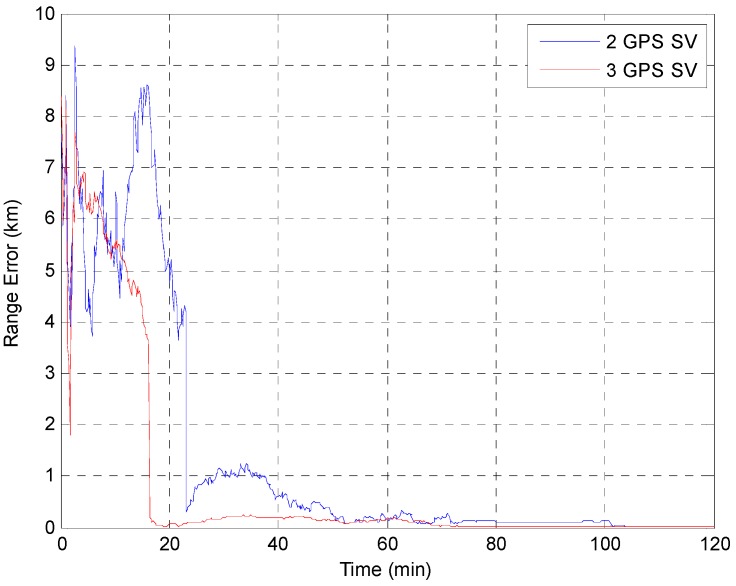
Test result of the EKF simulation started at point A.

**Figure 15:**
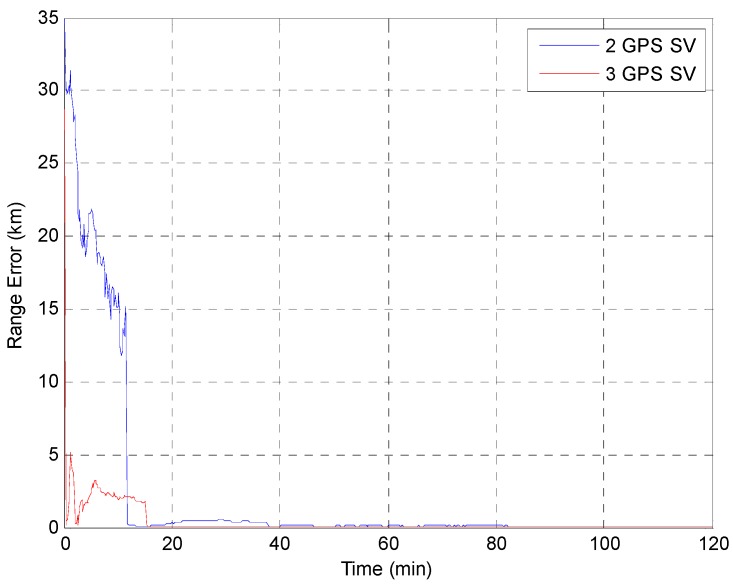
Test result of EKF simulation started at point B.

**Figure 16:**
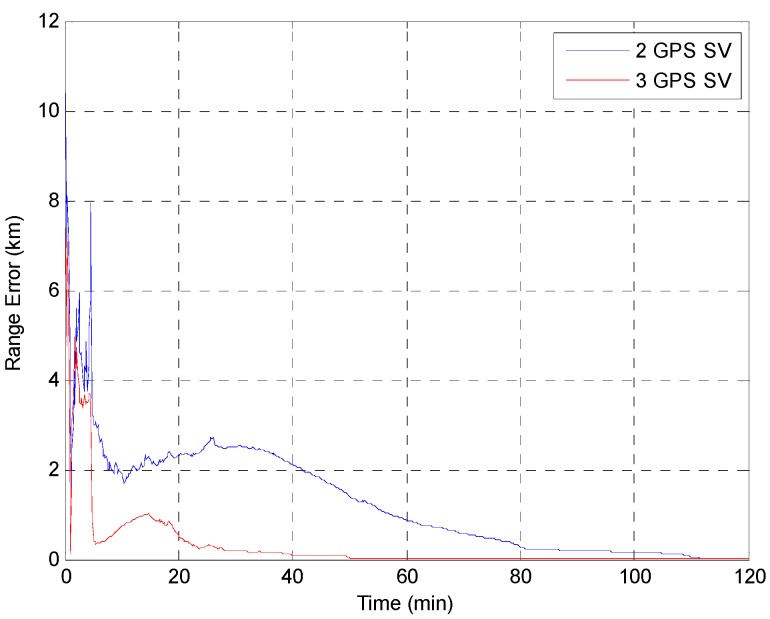
Test result of the EKF simulation started at point C.

**Figure 17:**
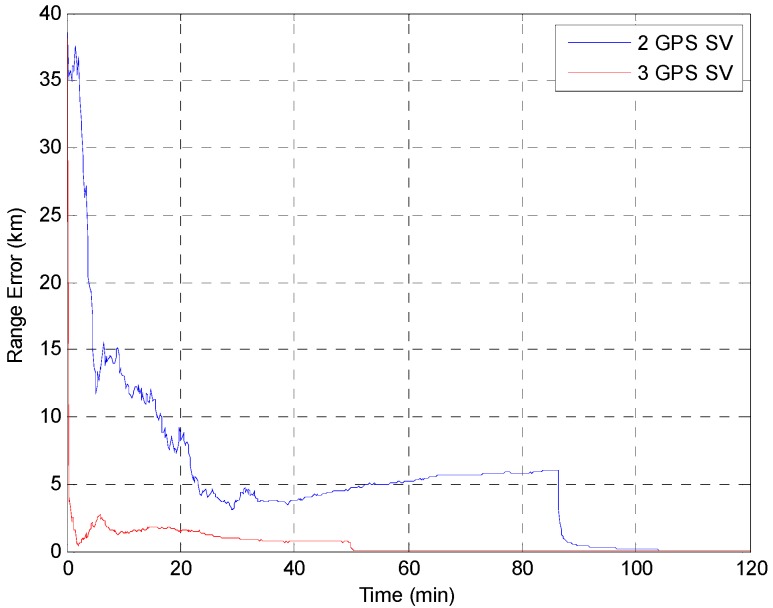
Test result of the EKF simulation started at point D for GPS satellite position D1.

**Figure 18:**
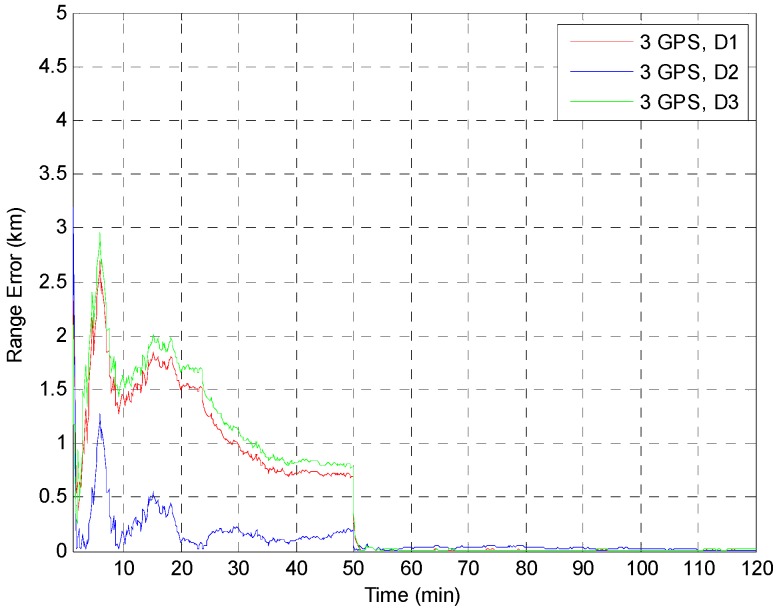
Test result of the EKF simulations started at point D for various positions of the third GPS satellite. The *Y* axis is zoomed for convenience.

**Figure 19:**
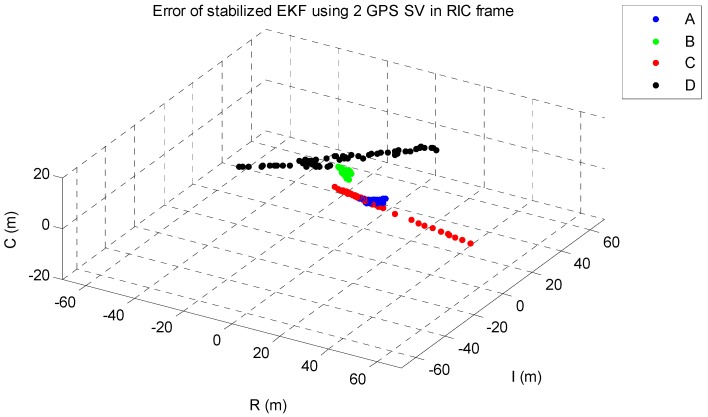
The stabilized EKF errors in a RIC frame when using two GPS satellites. (R is radial, I is along-track and C is cross-track direction).

**Figure 20:**
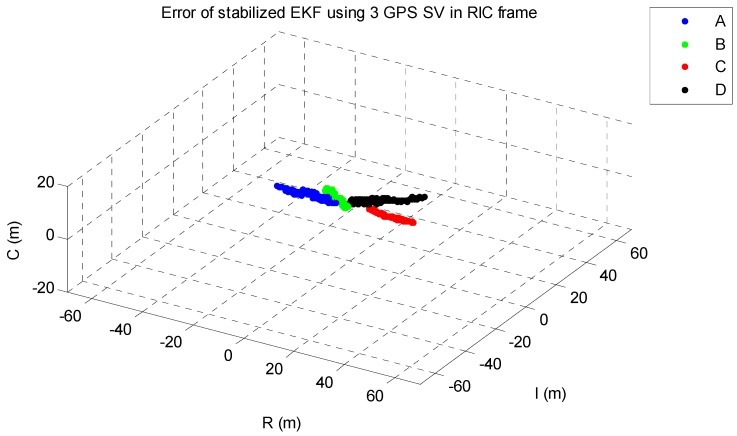
The stabilized EKF errors in a RIC frame when using three GPS satellites.

The EKF was tested using the initial state vector calculated by the developed algorithm at each point. The time update was processed using the Runge-Kutta method. The simulation results of the EKF are shown in Figure 14, Figure 15, Figure 16, Figure 17 and Figure 18. The time for filter convergence varied across the simulation points and the error of the initial state; however, the filter converged within 120 min with an accuracy of 100 m in all simulations. The errors expressed in the RIC frame after the filter is stabilized are shown in Figure 19 and Figure 20, and the error norm is bounded at 30 m when using three GPS satellites. These results were quite acceptable because the EKF filter for a GEO converges very slowly due to the orbit’s characteristics. For example, the convergence time of the EKF filter for a GEO is approximately one or two hours under the sparse visibility of GPS satellites [15]. The convergence rate of the filter depends on the geometric location of the GPS satellites and the changing visibility of the GPS satellites, and thus, the convergence rate varies even though the accuracies of the initial conditions are not significantly different.

## 5. Conclusions

The main goal of the algorithm is to calculate the state of the GEO satellite using a single-epoch measurement under the sparse visibility of the GPS satellites, which is usually impossible, even when applying the classical SPS algorithm. The proposed algorithm can calculate the position, velocity and receiver’s clock bias using only a single-epoch measurement of two or three GPS satellites without data from external sources. Therefore, the calculated result can be used as the initial state as soon as the measurements are generated by the receiver. The resulting maximum range error is less than 40 km, and when using the result as the initial state of the EKF, which uses a very accurate dynamic model, the filter converges to an error of 100 m within 120 min in the worst case. The maximum error norm of the EKF after filter stabilization is bounded at 30 m when three GPS satellites are observable. Given this acceptable result and the benefits of the algorithm, we expect that our algorithm is sufficiently accurate, robust, efficient, and practical for determining the initial orbit of GEO satellites.
